# Dexrazoxane Protects Cardiomyocyte from Doxorubicin-Induced Apoptosis by Modulating miR-17-5p

**DOI:** 10.1155/2020/5107193

**Published:** 2020-03-01

**Authors:** Xiaoxue Yu, Yang Ruan, Tao Shen, Quan Qiu, Mingjing Yan, Shenghui Sun, Lin Dou, Xiuqing Huang, Que Wang, Xiyue Zhang, Yong Man, Weiqing Tang, Zening Jin, Jian Li

**Affiliations:** ^1^The Key Laboratory of Geriatrics, Beijing Institute of Geriatrics, Beijing Hospital, National Center of Gerontology, National Health Commission, Institute of Geriatric Medicine, Chinese Academy of Medical Sciences, Beijing 100730, China; ^2^Peking University Fifth School of Clinical Medicine, Beijing 100730, China; ^3^Beijing Tiantan Hospital, Capital Medical University, 100070 Beijing, China; ^4^Department of Emergency Cardiology, Beijing Anzhen Hospital, Capital Medical University, Beijing 100029, China

## Abstract

The usage of doxorubicin is hampered by its life-threatening cardiotoxicity in clinical practice. Dexrazoxane is the only cardioprotective medicine approved by the FDA for preventing doxorubicin-induced cardiac toxicity. Nevertheless, the mechanism of dexrazoxane is incompletely understood. The aim of our study is to investigate the possible molecular mechanism of dexrazoxane against doxorubicin-induced cardiotoxicity. We established a doxorubicin-induced mouse and cardiomyocyte injury model. Male C57BL/6J mice were randomly distributed into a control group (Con), a doxorubicin treatment group (DOX), a doxorubicin plus dexrazoxane treatment group (DOX+DEX), and a dexrazoxane treatment group (DEX). Echocardiography and histology analyses were performed to evaluate heart function and structure. DNA laddering, qRT-PCR, and Western blot were performed on DOX-treated cardiomyocytes with/without DEX treatment in vitro. Cardiomyocytes were then transfected with miR-17-5p mimics or inhibitors in order to analyze its downstream target. Our results demonstrated that dexrazoxane has a potent effect on preventing cardiac injury induced by doxorubicin in vivo and in vitro by reducing cardiomyocyte apoptosis. MicroRNA plays an important role in cardiovascular diseases. Our data revealed that dexrazoxane could upregulate the expression of miR-17-5p, which plays a cytoprotective role in response to hypoxia by regulating cell apoptosis. Furthermore, the miRNA and protein analysis revealed that miR-17-5p significantly attenuated phosphatase and tensin homolog (PTEN) expression in cardiomyocytes exposed to doxorubicin. Taken together, dexrazoxane might exert a cardioprotective effect against doxorubicin-induced cardiomyocyte apoptosis by regulating the expression of miR-17-5p/PTEN cascade.

## 1. Introduction

The incidence of cancer has increased in recent years, and it is speculated that 13.1 million people will die of cancer in 2030 [1]. Doxorubicin (DOX), an anthracycline antibiotic, is deemed to be one of the most effective frontline chemotherapeutic drugs for treating cancers [2]. While doxorubicin has a broad-spectrum anticancer activity, the severe adverse effects, especially life-threatening cardiotoxicity, limit its clinical application [3]. Free radical-mediated myocytes damage is the first and most thoroughly studied mechanism used to explain doxorubicin-induced cardiotoxicity [4]. Excess ROS could result in DNA damage and cardiomyocyte apoptosis [5]. Nevertheless, the precise molecular mechanism of the doxorubicin-induced cardiomyocyte apoptosis still remains poorly defined.

MicroRNAs (miRNAs) are a class of noncoding RNAs about 22 nucleotides in length, which are reported to posttranscriptionally regulate target gene expression by directly binding to 3′-untranslated regions (3′-UTR) of target messenger RNAs [6]. It has been well recognized that a large number of miRNAs participate in regulating doxorubicin-induced cardiotoxicity; thus, they could be used as potential cardiotoxicity biomarkers [7]. MiR-17-5p belongs to miR-17 family, which has been confirmed to be involved in the normal development of organisms and the survival and growth of malignant tumor [8]. A study reported that overexpression of miR-17-5p could suppress the inflammation in LPS-induced macrophages [9]. Furthermore, it has been found that miR-17-5p plays the role of oncogene in most tumors, promotes cell proliferation, and inhibits cell apoptosis [10, 11]. Moreover, the recent study has shown that miR-17-5p is downregulated in breast cancer patients with epirubicin- (an isomer) induced cardiotoxicity [12]. Based on these findings, we postulate that miR-17-5p may take part in the regulation of doxorubicin-induced cardiotoxicity.

Dexrazoxane (DEX) is the only cardioprotective medicine approved by FDA for preventing anthracycline-induced cardiac toxicity [13]. Numerous studies have proved that dexrazoxane could chelate iron to decrease the generation of ROS, thus preventing ROS-induced cardiomyocyte apoptosis [14, 15]. However, no research that has focused on miRNAs concerning the cardioprotective effect of dexrazoxane.

In this study, we aim to investigate the molecular mechanism of the protective role of dexrazoxane in doxorubicin-induced cardiotoxicity and to determine whether miRNAs are involved in this protective effect.

## 2. Materials and Methods

### 2.1. Regents and Antibodies

Dulbecco's Modified Eagle Medium (DMEM) (high glucose), Trypsin-EDTA, Thiazolyl Blue Tetrazolium Bromide, Protease Inhibitor Cocktail and Phosphatase Inhibitor Cocktail 3, and Dimethyl Sulfoxide (DMSO) were purchased from Sigma-Aldrich (Sigma, USA). Protein concentration was determined by BCA protein assay kit from Pierce (Rockford, AL). Spectra Multicolor Broad Range Protein Ladder were purchased form Thermo Scientific Hyclone (Hyclone, USA). Fetal Bovine Serum (FBS) and antibiotic penicillin/streptomycin were obtained from Gibco (Gibco, Invitrogen). Cell Lysis Buffer (10x), antibodies directed against Bax, caspase 3, PTEN, NF-*κ*B, p38MAPK, phosphorylated-NF-*κ*B, phosphorylated-p38MAPK, GAPDH, and Goat Anti-Rabbit IgG-HRP were from Cell Signaling Technology (Beverly, MA).

### 2.2. Animal Model

Male C57BL/6J mice (18-22 g, 10 weeks old) were purchased from SPF (Beijing) Biotechnology Co. All animal experiments were carried out according to the Guide for Care and Use of Laboratory Animals (NIH Publication # 85-23, revised 1996). The mice (*n* = 32) were randomly distributed into a control group (Con), a doxorubicin treatment group (DOX), a doxorubicin plus dexrazoxane treatment group (DOX+DEX), and a dexrazoxane treatment group (DEX). DOX+DEX mice were pretreated with 0.1 ml dexrazoxane solutions (200 mg/kg/day, dissolved in 0.167 mol/l sodium lactate solution) 1 h before 10 mg/kg doxorubicin treatment three times a week. DOX mice were injected with the same volume sodium lactate solution and doxorubicin. DEX mice were injected with the same volume dexrazoxane and saline. Con mice were injected with the same volume sodium lactate solution and saline. All of the mice in the four groups were euthanized 7 days after the initial injection of doxorubicin, and the dose of doxorubicin was modified according to previous studies [16–20].

### 2.3. Cardiac Function Assessment

Echocardiography was measured using Vevo 770 and Vevo 2100 (VisualSonics) instruments from Peking University Third Hospital. Fraction shortening (FS) and ejection fraction (EF) were assessed with Vevo Analysis software (version 2.2.3) as previously described [21]. After echocardiography examination, mice were euthanatized by cervical dislocation, and the hearts were collected for cardiac histological analysis.

### 2.4. Cardiac Histological Analysis

Histology assays were performed with hearts and sections as previously described [22]. The mouse heart tissues were collected and fixed with 4% paraformaldehyde. Tissues were processed as paraffin section and subsequently analyzed by hematoxylin-eosin staining according to the manufacturer's protocol (Sigma-Aldrich). The sections were imaged by microscopy.

### 2.5. Cardiomyocyte Isolation and Culture

Cardiomyocytes were isolated from 1- to 3-day-old C57BL/6J mice. The mouse hearts were digested with the combination of trypsin and collagenase type II. The detailed operation procedure was as previously described [23]. Cardiomyocytes were seeded at a density of 6.6 × 10^4^ cells/cm^2^ and cultured in DMEM supplemented with 10% FBS, 100 U/ml penicillin, 100 *μ*g/ml streptomycin, and 0.1 mM 5-bromo-2-deoxyuridine (Sigma-Aldrich, USA). Cells were maintained at 37°C in a humidified atmosphere with 5% CO2 and 95% air (v/v).

### 2.6. MTT Assay

Cardiomyocyte viability was determined with MTT assay kit (3-(4, 5-dimethylthiazol-2-yl)-2, 5-diphenyltetrazolium bromide; Sigma-Aldrich, USA) according to the instructions. Cardiomyocytes were plated in 96-well plates and pretreated with or without dexrazoxane 1 h before treated with or without doxorubicin. 0.5 mg/ml 3-(4, 5-dimethylthiazol-2-yl)-2, 5-diphenyltetrazolium bromide was added to the cells. Four hours after, the absorbance was detected at 490 nm.

### 2.7. LDH Assay

The lactate dehydrogenase (LDH) concentration was measured using an LDH assay kit according to the manufacturer's manual (Solarbio, Beijing, China) by a routine microtitre plate reader (wavelength: 572 nm).

### 2.8. Hoechst33342 Staining

Nuclear condensation was detected by Hoechst staining. Cardiomyocytes were fixed with 4% PFA for 10 min. Then washed the cells and incubated them in 10 mM Hoechst 33342 (Sigma-Aldrich) in the dark for 5 min. The cells were viewed using a fluorescence microscope with a blue/cyan emission filter as described previously [24].

### 2.9. DNA Ladder Assay

Cells were lysed in lysis buffer (10 mM Tris-Cl pH 8.0, 150 mM NaCl, 0.4% SDS, 10 mM EDTA, and 100 g/ml protease K) and incubated at 37°C overnight with gentle agitation. DNA was extracted with phenol/CHCl3/isoamyl alcohol once and CHCl3/isoamyl alcohol twice. DNA fragmentation was detected by loading 10 *μ*g of total DNA onto 2% agarose gel in Tris acetate/EDTA buffer and visualized by ethidium bromide staining as described previously [24].

### 2.10. Western Blotting Analysis

The protein was extracted from mouse hearts and cardiomyocytes were lysed with cell lysis buffer supplemented with protease and phosphatase inhibitors. The concentration of protein was measured by Pierce BCA Protein Assay Kit. Equivalent protein (10-20 *μ*g) was electrophoresed on sodium dodecyl sulfate-polyacrylamide gels (12%) and transferred to polyvinylidene fluoride (PVDF) membranes. The members were blocked with 5% nonfat milk for 2 h at room temperature. Then the membranes were probed with specific first antibodies(1 : 1000) at 4°C overnight, including the following antibodies:caspase3, Bax, PTEN, NF-*κ*B, p38MAPK, phosphorylated-NF-*κ*B, phosphorylated-p38MAPK, and GAPDH. Membranes were then incubated with secondary antibody (1 : 5000) for 2 h at room temperature. The signals were visualized with an ECL detection reagent. Densitometry analysis was then performed with Image J software (V1.8.0.112).

### 2.11. Cell Transfection

miRNA oligos were purchased from GenePharma Biotech (Shanghai). Cardiomyocytes were transferred with miR-17-5p mimics, mimics negative control, miR-17-5p inhibitors, or inhibitors negative control using Lipofectamine RNAiMAX (Invitrogen, USA) following the directions provided by the manufacturer. After 24 h, the cells were treated with or without dexrazoxane and doxorubicin for another 24 h.

### 2.12. RNA Extraction and Real-Time Quantitative PCR

The total RNA was isolated using TRIzol (Invitrogen, Carlsbad, CA, USA). Reverse transcription was performed using a kit from New England Biolabs. The levels of mature miRNA were performed using the QuantStudio3 Real-Time PCR system (Thermo Fisher Scientific, US) with SYBR Green (TaKaRa, Japan) according to the instructions. The data were presented at least three independent experiments.

### 2.13. 3′UTR Luciferase Assays

The 3′-untranslated region (3′-UTR) of target genes and their mutant variant were synthesized and digested with SacI and XhoI to generate reporter vectors containing miRNA-binding sites (Shengong Co., China). HEK-293A cells were seeded in 96-well plates and cotransfected with luciferase reporter and miR-17-5p mimics using a transfection reagent (Vigofect, Vigorous Biotechnology, China). The cells were harvested 48 h later, and the luciferase activity was detected using the Dual-Luciferase Reporter Assay System (Promega).

### 2.14. Statistics

All results were analyzed by GraphPad Prism 6 software (GraphPad Software, CA, USA). The data were presented as the mean ± standard error of mean (SEM). Statistical comparisons between two groups were performed by Student's *t*-test, and among multiple groups were performed by one-way ANOVA followed by a Bonferroni correction. *P* < 0.05 was considered statistically significant.

## 3. Results

### 3.1. Dexrazoxane Mitigates Doxorubicin-Induced Cardiac Injury In Vivo

**Figure 1:**
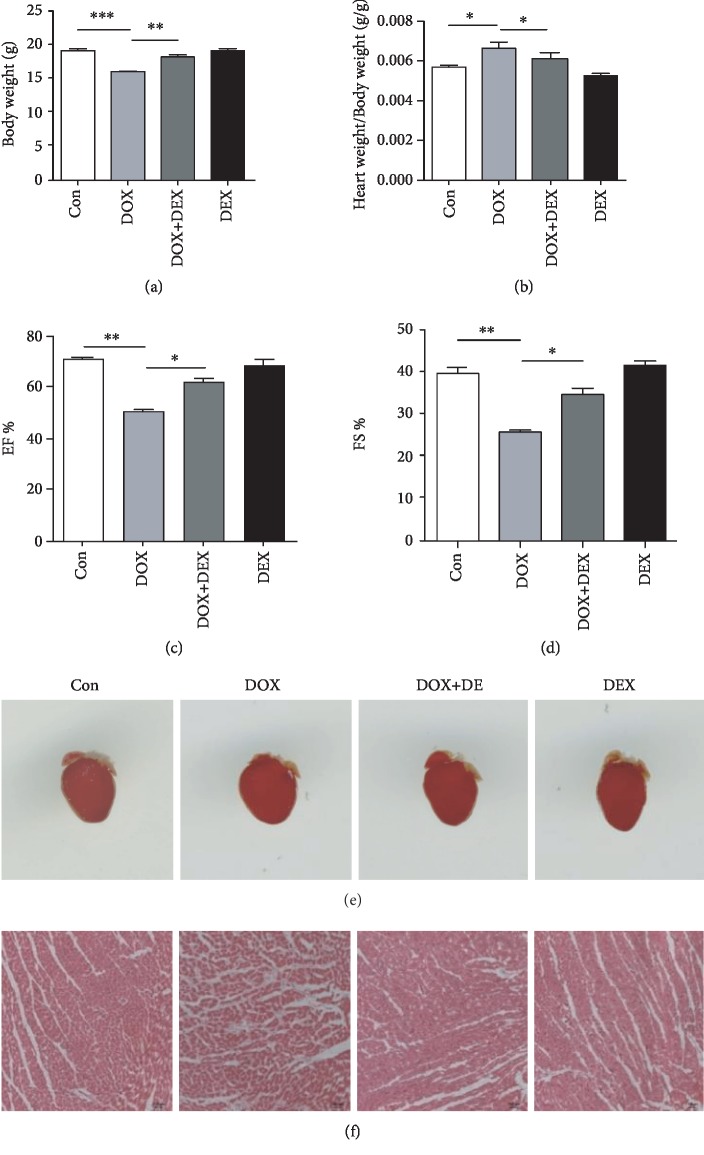
Dexrazoxane mitigates doxorubicin-induced cardiac injury in vivo. (a,b) Body weight and heart weight to body weight (HW/BW) ratio in control mice (Con), doxorubicin-treated mice (DOX), doxorubicin plus dexrazoxane-treated mice (DOX+DEX), and dexrazoxane-treated mice (DEX). (c,d) Ejection fraction % (EF %) and fractional shortening % (FS %) after 7 days doxorubicin treatment in Con, DOX, DOX+DEX, and DEX mice. (e,f) Representative images of mouse hearts and hematoxylin and eosin staining of the heart paraffin section of Con, DOX, DOX+DEX, and DEX groups (*n* = 8). (∗*P* < 0.05, ∗∗*P* < 0.01, ∗∗∗*P* < 0.001).

To explore the effect of dexrazoxane on doxorubicin-induced cardiotoxicity in vivo, we used doxorubicin-treated mice to establish a heart failure model. We observed that doxorubicin treatment resulted in significant decrease of body weight and increase of heart/body weight ratio compared with control mice, while dexrazoxane pretreatment could mitigate symptoms (Figures 1(a) and 1(b)). Echocardiography analysis of ejection fraction (EF) % and fraction shortening index (FS) % indicated that doxorubicin could induce heart function loss in vivo. However, dexrazoxane treatment could attenuate heart function loss significantly (Figures 1(c) and 1(d)). Hematoxylin-eosin staining showed that a large amount of inflammatory cells were accumulated in the heart tissue, and the structure of heart tissue was disordered in the doxorubicin (DOX) group compared with the control (Con) group. Nevertheless, dexrazoxane significantly decreased inflammatory cell accumulation and preserved the myocardial structure (Figures 1(e) and 1(f)). Together, these data suggested that dexrazoxane has a potent effect on preventing cardiac injury induced by doxorubicin.

### 3.2. Doxorubicin Decreases Cell Viability and Promotes Cardiomyocyte Apoptosis In Vitro

**Figure 2:**
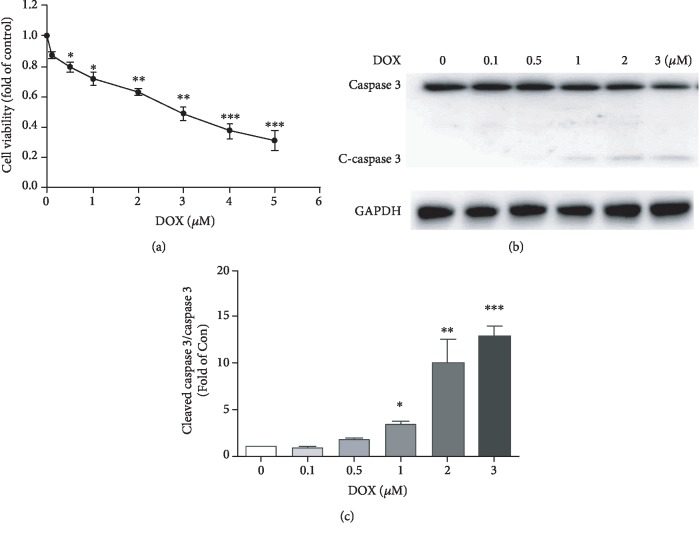
Doxorubicin causes cell injury by promoting apoptosis in cardiomyocytes. (a) The cell viability was measured by MTT assay in primary cardiomyocytes treated with different concentrations of doxorubicin (0, 0.1, 0.5, 1, 2, 3, 4, and 5 *μ*M) for 24 h (*n* = 4). (b,c) Western blotting analysis of caspase 3 and cleaved caspase 3 in cardiomyocytes cultured with different concentrations of doxorubicin (0, 0.1, 0.5, 1, 2, and 3 *μ*M) for 24 h (*n* = 3). (∗*P* < 0.05, ∗∗*P* < 0.01, ∗∗∗*P* < 0.001 vs the control group).

We used MTT method to measure the viability of primary cardiomyocytes after doxorubicin treatment. As shown in Figure 2(a), doxorubicin treatment reduced cardiomyocyte viability in a concentration-dependent manner. Western blotting analysis showed that active (cleaved) caspase 3 substantially increased in a dose-dependent manner after doxorubicin treatment in cardiomyocyte (Figures 2(b) and 2(c)). These results suggested that doxorubicin could reduce myocyte viability and induce cardiomyocyte apoptosis. The moderate injury for cardiomyocyte is 1 *μ*M doxorubicin, when cell viability declined about 30% and cleaved-caspase 3 significantly increased. Therefore, we used 1 *μ*M doxorubicin in the subsequent experiments to generate the in vitro doxorubicin-induced cardiomyocyte toxicity model.

### 3.3. Dexrazoxane Ameliorates Doxorubicin-Induced Cardiomyocyte Apoptosis

**Figure 3:**
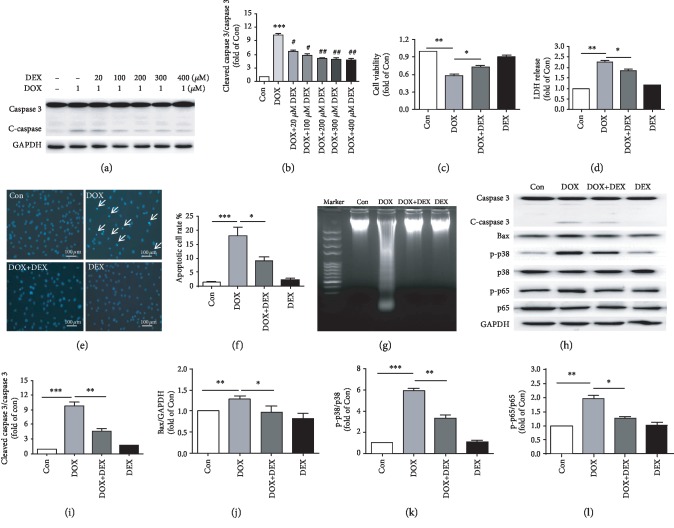
Dexrazoxane alleviates doxorubicin-mediated cytotoxicity and cardiomyocytes apoptosis. (a,b) The expression of caspase 3 and cleaved caspase 3 in cardiomyocytes pretreated with different concentrations of dexrazoxane (0, 20, 100, 200, 300, and 400 *μ*M) for 1 h, then treated with or without 1 *μ*M doxorubicin (*n* = 4, ∗∗∗*P* < 0.001 versus Con group; #*P* < 0.5, ##*P* < 0.01 versus DOX group). (c) The cell viability was estimated by MTT assay in Con, DOX, DOX+DEX, and DEX groups (*n* = 3). (d) LDH concentration in the medium after treated with or without dexrazoxane and doxorubicin (*n* = 3). (e,f) Hoechst staining for Con, DOX, DOX+DEX, and DEX groups (*n* = 4). (g) DNA laddering for Con, DOX, DOX+DEX, and DEX groups. (h–l) Caspase 3, cleaved-caspase 3, Bax, phosphorylated-p38MAPK, p38MAPK, phosphorylated-p65, p65, and GAPDH in cardiomyocyte treated with or without 200 *μ*M dexrazoxane and 1 *μ*M doxorubicin (*n* = 3). (∗*P* < 0.05, ∗∗*P* < 0.01, ∗∗∗*P* < 0.001).

Given that apoptosis plays an important role in doxorubicin-induced cardiotoxicity [25], we subsequently determined the effect of dexrazoxane on cardiomyocyte apoptosis. We treated cardiomyocytes with different concentrations of dexrazoxane prior to doxorubicin exposure. Western blotting showed that the expression of cleaved-caspase 3 in doxorubicin plus dexrazoxane- (DOX+DEX-) treated group was significantly lower than that of doxorubicin (DOX) alone. When the concentration is 200 *μ*M, the effect of dexrazoxane reached its peak (Figures 3(a) and 3(b)). Thus, we applied 200 *μ*M dexrazoxane to cardiomyocytes before doxorubicin treatment. The MTT assay showed that pretreatment with dexrazoxane could improve the cardiomyocyte viability, which was decreased by doxorubicin (Figure 3(c)). LDH, as another maker of cellular damage, was dramatically enhanced by doxorubicin and reduced obviously in DOX+DEX group (Figure 3(d)). Hoechst staining revealed that DNA condensation and fragmentation was induced by doxorubicin, which is an indicator of apoptosis. Nevertheless, the apoptotic cardiomyocytes were obviously reduced by dexrazoxane (Figures 3(e) and 3(f)). In addition, an occurrence of DNA laddering was discovered in doxorubicin-treated cardiac myocytes, which could be diminished by dexrazoxane (Figure 3(g)). Western blotting also revealed that active (cleaved) caspase 3 and Bax were decreased obviously in the DOX+DEX group compared with DOX group. Moreover, we found that doxorubicin treatment could increase the level of phosphorylated-p38MAPK and phosphorylated-p65, which is an important inflammation signaling pathways, while pretreatment with dexrazoxane could reverse this effect (Figures 3(h)–3(l)). Our study showed that dexrazoxane may protect doxorubicin-mediated cytotoxicity and apoptosis via p38MAPK/NF-*κ*B signaling pathway.

### 3.4. mir-17-5p Directly Targets PTEN by Interacting with Its 3′UTR

**Figure 4:**
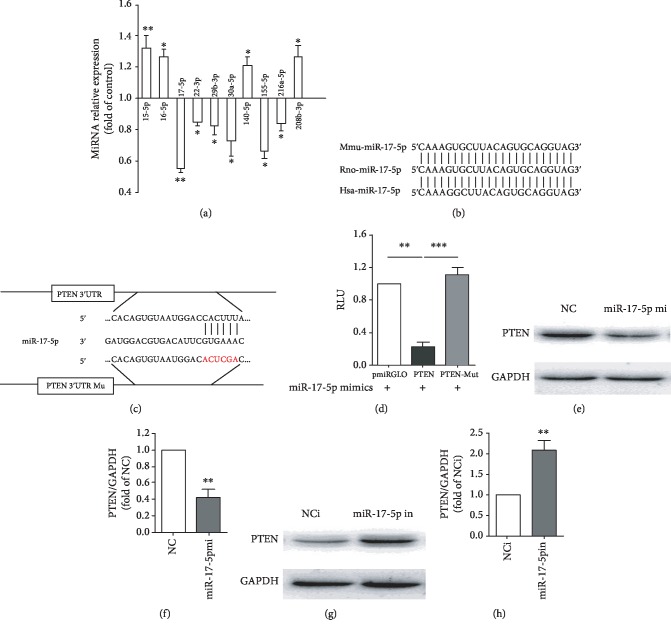
MiR-17-5p directly targets PTEN and regulates its expression. (a) The expression of miR-15-5p, miR-16-5p, miR-17-5p, miR-22-3p, miR-29b-3p, miR-30a-5p, miR-140-5p, miR-155-5p, miR-216a-5p, and miR-208b-3p were detected by qPCR in the Con and DOX groups (*n* = 4). (b) Conservation of the miR-17-5p sequence between mice, rats, and humans is shown. (c) A binding site for miR-17-5p in the 3′-UTR of PTEN was analyzed by target prediction programs, and the mutated sequence of the binging site is marked in red. (d) Luciferase activity was assessed in HEK-293A cells 24 h after transfection with the indicated plasmids: miR-17-5p+vectors, miR-17-5p+PTEN 3′UTR plasmid, and miR-17-5p+mutated PTEN 3′UTR plasmid (*n* = 4). (e,f) The level of PTEN was detected by Western blotting in normal control (NC) and miR-17-5p mimics groups (*n* = 3). (g,h) The expression of PTEN was estimated using Western blotting in normal control of inhibitor (NCi) and miR-17-5p inhibitor groups (*n* = 3). (∗*P* < 0.05, ∗∗*P* < 0.01).

The results in Figure 4(a) showed that several miRNA expressions were analyzed in doxorubicin-treated cardiomyocytes. MiR-17-5p expression change was most obvious in all the genes. Upon analyzing the sequence, miR-17-5p has been highly conserved in mouse, rat, and human (Figure 4(b)). Next, we tried to identify miR-17-5p direct downstream targets using the target prediction programs TargetScan, miRBase, and PicTar. We analyzed 8 candidate genes with miR-17-5p binding sites in their 3′-UTRs. We synthesized the wild type 3′-UTR and mutated binding sites 3′-UTR of each candidate gene into a pmiRGLO vector and did the dual-luciferase reporter assay to verify whether miR-17-5p directly bounds to these genes. Our data indicated that the phosphatase and tensin homolog (PTEN), an apoptosis-related gene, is a potential molecular target of miR-17-5p in cardiomyocyte (Figure 4(c)). To investigate miR-17-5p's direct target gene, we cotransfected miR-17-5p mimics with the luciferase reporters into HEK293A cells. The relative luciferase activity of the PTEN 3′-UTR reporter was obviously declined compared with the control vector. However, when the miR-17-5p binding site in the 3′-UTR of PTEN was mutated, the relative luciferase activity of the PTEN 3′-UTR reporter was consistent with the control vector (Figure 4(d)). Western blotting showed that PTEN expression was significantly decreased after miR-17-5p mimic transfection and increased with the miR-17-5p inhibitor transfection in cardiomyocytes (Figures 4(e)–4(h)). Thus, the data suggest that miR-17-5p could directly bind to PTEN 3′-UTR and inhibit its expression.

### 3.5. Dexrazoxane Upregulates miR-17-5p Level and Protects Cardiomyocytes against Doxorubicin-Induced Apoptosis

**Figure 5:**
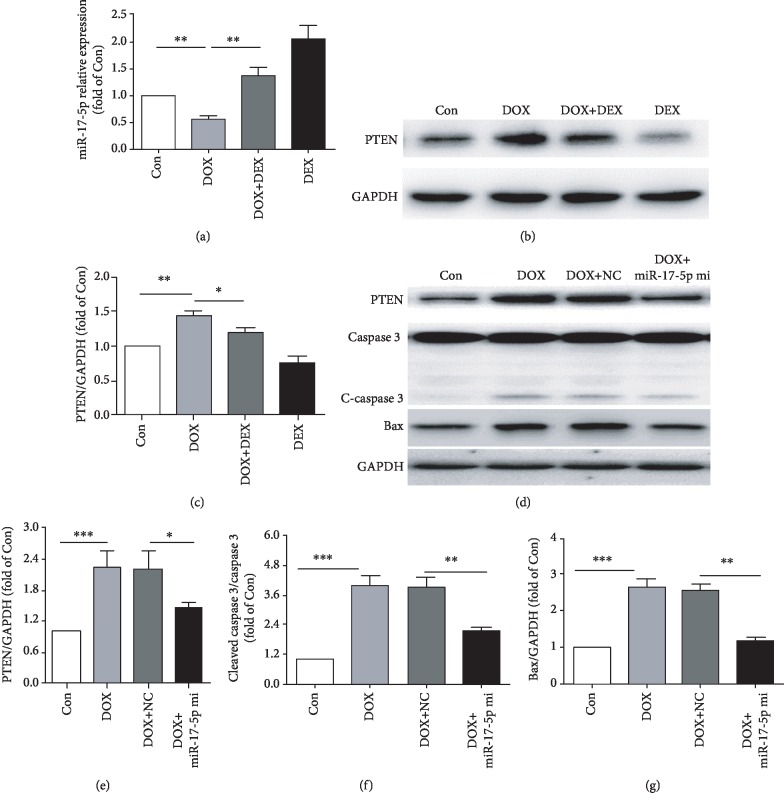
Dexrazoxane attenuates doxorubicin mediated cytotoxicity by the regulation of miR-17-5p/PTEN cascade. (a) The expression of miR-17-5p was demonstrated by qPCR in the Con, DOX, DOX+DEX, and DEX groups (*n* = 4). (b,c) Western blotting and average data for PTEN in Con, DOX, DOX+DEX, and DEX groups (*n* = 3). (d–g) Protein level of PTEN, caspase3, cleaved-caspase3, and Bax in Con, DOX, DOX+NC, and DOX+miR-17-5p mimics groups (*n* = 4). (∗*P* < 0.05, ∗∗*P* < 0.01, ∗∗∗*P* < 0.001).

To investigate the protective molecular mechanism of dexrazoxane on doxorubicin-induced cardiotoxicity, we detected the miR-17-5p expression in cardiomyocytes. The results in Figure 5(a) presented that doxorubicin notably downregulated the expression levels of miR-17-5p. Interestingly, dexrazoxane could significantly promote miR-17-5p expression in doxorubicin-treated cardiomyocytes. Western blotting showed that PTEN protein level was upregulated in doxorubicin-treated cardiomyocytes, which could be lowered by dexrazoxane (Figures 5(b) and 5(c)). Moreover, overexpression of miR-17-5p could reduce the increase in PTEN caused by doxorubicin and decrease the expression of cleaved caspase 3 and Bax (Figures 5(d)–5(g)). Previous studies have stated that repression of PTEN could mitigate hypoxia-induced cardiomyocyte apoptosis (18). Taken together, the results indicated that dexrazoxane might protect cardiomyocytes from doxorubicin-induced apoptosis by regulating miR-17-5p/PTEN pathways.

## 4. Discussion

In the present study, we demonstrated that dexrazoxane protects heart function and prevents doxorubicin-triggered apoptosis. Additionally, dexrazoxane could inhibit doxorubicin-induced cardiomyocyte apoptosis by regulating the expression of miR-17-5p/PTEN cascade. As far as we know, this is the first report to show that dexrazoxane may protect cardiomyocyte from doxorubicin-induced injury by modulating miRNA expression and its downstream signal pathway.

Apoptosis is known as type I cell death, which plays a key important role in the development and progression of cardiovascular disease [26–28]. Apoptosis could be initiated via extrinsic and intrinsic pathways [29]. A large number of studies have proved that cardiomyocyte apoptosis is a vital feature of doxorubicin-induced cardiotoxicity [30]. Consistent with the previous studies [31, 32], we observed a significant decrease of heart function in doxorubicin-treated mice, accompanied with an increase of heart/body weight ratio. Moreover, our in vitro experiments confirmed that doxorubicin could reduce cell viability and cause cardiomyocyte apoptosis, evidenced by increasing the expression of cleaved-caspase 3 and Bax.

As mentioned above, dexrazoxane is the only cardioprotective drug approved by the FDA to resist doxorubicin-induced cardiac damage [13]. However, the exact protective mechanism of dexrazoxane remains elusive. Multiple mechanisms have been proposed to contribute the protective effects of dexrazoxane. Some researches revealed that dexrazoxane could chelat iron to reduce the generation of ROS [4, 33]. Bures et al. [34] found that dexrazoxane could prevent doxorubicin from binding to the topoisomerase 2*β* complex. Our results showed that dexrazoxane improved cardiac function and blocked cardiomyocyte apoptosis, which was in line with the results of Suzuki et al. [35].

The cardiotoxicity induced by doxorubicin has been associated with inflammatory cytokines, many of which are modulated by mitogen-activated protein kinases (MAPKs) [36]. Many studies have demonstrated that doxorubicin could increase the expression of phosphorylated-p38MAPK and phosphorylated-NF-*κ*B, which play an important role in activation of inflammation [37, 38]. The research of Thandavarayan et al. showed that Schisandrin B could prevent doxorubicin-induced inflammation through inhibition of p38MAPK signaling [39]. In addition to regulating inflammation, p38MAPK can also control cell cycle and apoptosis. Ni et al. and Shati et al. [40, 41] found that doxorubicin could promote the activation of p38MAPK, thus promoting the occurrence of apoptosis . Similarly, we also found that doxorubicin could active the p38 MAPK and NF-*κ*B, while dexrazoxane could repress this process. These results supposed that dexrazoxane could protect doxorubicin-induced apoptosis and inflammation by p38MAPK/NF-*κ*B signaling pathway.

There has been a great number of evidence supporting that miRNAs may take part in doxorubicin-induced cardiotoxicity [42–44]. Moreover, previous studies have manifested that miR-17-5p plays a vital role in tumor cell survival, and it has antiapoptotic properties [45, 46]. In this study, the level of miR-17-5p was markedly declined in doxorubicin-treated cardiomyocytes, which could be recovered by dexrazoxane, and overexpression of miR-17-5p could reduce doxorubicin-induced apoptosis in cardiomyocytes.

PTEN (phosphatase and tensin homolog on chromosome 10) is a tumor suppressor, which dephosphorylates PIP3, thereby inhibiting the AKT/mTOR pathway [47]. Hu et al. [48] found that decreasing miR-21 could exert antiapoptotic effect by targeting PTEN in rats. In addition, Yuan et al. [49] determined that miR-19b and miR-20a may suppress myeloma cells apoptosis by targeting PTEN. Our studies also confirmed that PTEN is a pro-apoptosis gene. Fang et al. [50]reported that miR-17-5p could induce drug resistance an invasion of ovarian carcinoma cells by targeting PTEN signaling. Lu et al. [51] discovered that long noncoding RNA HOTAIRMI inhibits cell progression by regulating miR-17-5p/PTEN axis in gastric cancer. Consistent with the previous studies, in our work, we verified that PTEN is the target gene of miR-17-5p, and dexrazoxane might alleviate doxorubicin-triggered apoptosis via miR-17-5p/PTEN signal pathway.

## 5. Conclusions

In this study, we proved that dexrazoxane prevents doxorubicin-induced cardiotoxicity by ameliorating apoptosis. We demonstrate for the first time that miR-17-5p plays a key role in the cardioprotective effect of dexrazoxane, which may help to better understand the cardioprotection of dexrazoxane, and miR-17-5p could be a potential molecular target in doxorubicin-induced cardiotoxicity treatment in the future. Moreover, the present findings may offer a new insight to implicate novel drug targets and offer new therapeutic strategies to protect against doxorubicin-induced cardiotoxicity.

## Data Availability

The datasets used and/or analyzed during the current study are available from the corresponding author on reasonable request.

## Supplementary Materials

Supplementary Materials Table 1: primers for reverse transcription PCR. The reverse transcription primer sequences of U6, miR-15b-5p, miR-16-5p, miR-17-5p, miR-22-3p, miR-29b-3p, miR-30a-5p, miR-140-5p, miR-155-5p, miR-216a-5p, and miR-208b-3p. Table 2: primers for Quantitative Real-time PCR. The Quantitative real-time primer sequences of U6, miR-15b-5p, miR-16-5p, miR-17-5p, miR-22-3p, miR-29b-3p, miR-30a-5p, miR-140-5p, miR-155-5p, miR-216a-5p, and miR-208b-3p. Table 3: the siRNA of miR-17-5p. The sequences of MiR-17-5p mimics, miR-17-5p negative control of mimics, miR-17-5p inhibitor, and miR-17-5p inhibitor negative control.
