# Supplementary material for: Dexrazoxane Protects Cardiomyocyte from Doxorubicin-Induced Apoptosis by Modulating miR-17-5p
Source: Biomed Res Int. 2020 Mar 1;2020:5107193. doi: 10.1155/2020/5107193 (PMC7071803; doi:10.1155/2020/5107193)
Supplement: Supplementary Materials — Table 1: primers for reverse transcription PCR. The reverse transcription primer sequences of U6, miR-15b-5p, miR-16-5p, miR-17-5p, miR-22-3p, miR-29b-3p, miR-30a-5p, miR-140-5p, miR-155-5p, miR-216a-5p, and miR-208b-3p. Table 2: primers for Quantitative Real-time PCR. The Quantitative real-time primer sequences of U6, miR-15b-5p, miR-16-5p, miR-17-5p, miR-22-3p, miR-29b-3p, miR-30a-5p, miR-140-5p, miR-155-5p, miR-216a-5p, and miR-208b-3p. Table 3: the siRNA of miR-17-5p. The sequences of MiR-17-5p mimics, miR-17-5p negative control of mimics, miR-17-5p inhibitor, and miR-17-5p inhibitor negative control. [file 5107193.f1.docx]

**Supplemental materials**

**Table1：Primers for reverse transcription PCR.**

| **Reverse transcription primer name** | **Primer sequences** |
| --- | --- |
| U6 | GTCGTATCCAGTGCAGGGTCCGAGGTATTCGCACTGGATACGACAAATATG |
| miR-15b-5p | GTCGTATCCAGTGCAGGGTCCGAGGTATTCGCACTGGATACGACTGTAAAC |
| miR-16-5p | GTCGTATCCAGTGCAGGGTCCGAGGTATTCGCACTGGATACGACCGCCA |
| miR-17-5p | GTCGTATCCAGTGCAGGGTCCGAGGTATTCGCACTGGATACGACCTACCTG |
| miR-22-3p | GTCGTATCCAGTGCAGGGTCCGAGGTATTCGCACTGGATACGACACAGTTC |
| miR-29b-3p | GTCGTATCCAGTGCAGGGTCCGAGGTATTCGCACTGGATACGACAACACTG |
| miR-30a-5p | GTCGTATCCAGTGCAGGGTCCGAGGTATTCGCACTGGATACGACCTTCCA |
| miR-140-5p | GTCGTATCCAGTGCAGGGTCCGAGGTATTCGCACTGGATACGACCTACCATAG |
| miR-155-5p | GTCGTATCCAGTGCAGGGTCCGAGGTATTCGCACTGGATACGACACCCCT |
| miR-216a-5p | GTCGTATCCAGTGCAGGGTCCGAGGTATTCGCACTGGATACGACTCACAG |
| miR-208b-3p | GTCGTATCCAGTGCAGGGTCCGAGGTATTCGCACTGGATACGACACAAACC |

Table 1. The reverse transcription primer sequences of U6, miR-15b-5p, miR-16-5p, miR-17-5p, miR-22-3p, miR-29b-3p, miR-30a-5p, miR-140-5p, miR-155-5p, miR-216a-5p and miR-208b-3p.

**Table 2 ：Primers for Quantitative Real-time PCR**

| **Primer name** | **Primer sequences** |
| --- | --- |
| U6 5’ primer | GCGCGTCGTGAAGCGTTC |
| miR-15b-5p 5’ primer | ATCATCATTAGCAGCACATCATG |
| miR-16-5p 5’ primer | GGTCGTAGCAGGGCGTAAATA |
| miR-17-5p 5’ primer | CACATCATCAAAGTGCTTACAGTG |
| miR-22-3p 5’ primer | GCGTAAGCTGCCAGTTGAA |
| miR-29b-3p 5’ primer | GCGCTAGCACCATTTGAAAT |
| miR-30a-5p 5’ primer | GCGCGTGTAAACATCCTCGACTGG |
| miR-140-5p 5’ primer | CACATCATCATCAGTGGTTTTACC |
| miR-155-5p 5’ primer | CGCCTGTTAATGCTAATTGTGA |
| miR-216a-5p 5’ primer | ACGGGCTAATCTCAACTGGCAA |
| miR-208b-3p 5’ primer | GCCGGATAAGACGAACAAAA |
| 3’ Universal primer | GTGCAGGGTCCGAGGT |

Table 2. The Quantitative real-time primer sequences of U6, miR-15b-5p, miR-16-5p, miR-17-5p, miR-22-3p, miR-29b-3p, miR-30a-5p, miR-140-5p, miR-155-5p, miR-216a-5p and miR-208b-3p.

**Table3: The siRNA of miR-17-5p.**

| **Target regions** | **Sequences (5' to 3')** |
| --- | --- |
| miR-17-5p mimics | CAAAGUGCUUACAGUGCAGGUAG  ACCUGCACUGUAAGCACUUUGUU |
| negative control mimics | UUCUCCGAACGUGUCACGUTT  ACGUGACACGUUCGGAGAATT |
| miR-17-5p inhibitor | CUACCUGCACUGUAAGCACUUUG |
| inhibitor negative control | CAGUACUUUUGUGUAGUACAA |

Table 3. The sequences of MiR-17-5p mimics, miR-17-5p negative control of mimics, miR-17-5p inhibitor, miR-17-5p inhibitor negative control.
